# Lessons learned from a pandemic: implications for a combined exercise and educational programme for medical students

**DOI:** 10.1186/s12909-022-03290-1

**Published:** 2022-04-08

**Authors:** Aubree Worobetz, Andrew O’ Regan, Monica Casey, Peter Hayes, Mike O’ Callaghan, Jane C. Walsh, Enrique García Bengoechea, Catherine Woods, Deirdre McGrath, Liam G. Glynn

**Affiliations:** 1 School of Medicine and Health Research Institute, University of Limerick, Limerick, Ireland; 2 HRB Primary Care Clinical Trials Network, Galway, Ireland; 3 School of Psychology, National University of Ireland, Galway, Ireland; 4 Physical Activity for Health Research Cluster, Department of Physical Education and Sport Sciences, University of Limerick, Limerick, Ireland

**Keywords:** Physical activity, Exercise, Online, COVID-19

## Abstract

**Background:**

The ‘MED-WELL’ programme is a combined exercise and educational intervention designed to promote well-being among medical students and educate students about prescribing exercise as medicine in clinical practice. Due to COVID-19 public health restrictions of social distancing the ‘MED-WELL’ programme was offered online instead of in-person in 2021. The aim of this study is to compare the experiences of participants in the ‘MED-WELL’ programme online to those that previously participated in the same programme in-person to understand the student experience and optimize programme delivery.

**Methods:**

Purposive sampling was used to recruit 20 participants to a qualitative study using semi-structured interviews. Ten study participants took part in the ‘MED-WELL’ programme when it was offered in-person, and the other ten study participants took part in the programme when it was offered online. All interviews were audio-recorded and transcribed using Microsoft Teams. A combined inductive and deductive approach was used for analysis. An inductive thematic analysis was utilized to categorize data into higher order codes, themes, and overarching themes. The theory of online learning provided the theoretical framework for a deductive approach.

**Results:**

Analysis of the data produced five overarching themes: ‘student-student’, ‘student-teacher’, ‘student-content’, ‘student-environment’, and ‘effects of a pandemic’. The first four themes detail distinct types of interaction that participants had with various entities of the ‘MED-WELL’ programme and the effects that these interactions had on participant experiences. ‘Effects of a pandemic’ refers to the context of delivering the ‘MED-WELL’ programme online during a pandemic and how this mode of delivery influenced participants and the programme.

**Conclusions:**

Optimizing the ‘MED-WELL’ programme relies on an understanding of how participants interact with different entities of the programme and are motivated to attend and engage. Participants tended to favour an in-person mode of delivery, however certain advantages of delivering the programme online were also identified. The findings from this study can be used to inform similar experiential and educational exercise interventions, and may help plan for potential future restrictions on in-person educational and exercise-based programmes.

**Supplementary Information:**

The online version contains supplementary material available at 10.1186/s12909-022-03290-1.

## Background

Physical Activity (PA) is one of the most important behaviours for health and well-being, with benefits extending to both physical and mental health [1–3]. International PA guidelines recommend a minimum of 30 min of moderate to vigorous PA five days per week, or 150 min over a week to support physical and mental health [4]. Amongst medical students in particular, PA has a positive effect on mental and physical health, including reducing levels of anxiety, depression, and burnout [5–7].

In March 2020, the World Health Organization (WHO) declared the outbreak of coronavirus disease (COVID-19) a global pandemic. To inhibit its spread many national health authorities, including those in Ireland, recommended various public health measures including societal lockdown, restrictions on the gathering of groups of people, and physical distancing between people [8]. With these measures came public health recommendations such as closures of gymnasiums and fitness centers. This limited the ability to engage in PA and has affected PA levels worldwide [9]. A systematic review examining changes in PA during the pandemic reported an overall decrease in PA and increase in sedentary behaviour [10]. A study from November 2020 examined worldwide step-count data which also demonstrated a rapid decline in PA globally after the pandemic began [11]. In Ireland, about one in three people reported being less active than usual at the beginning of the pandemic [12].

In the context of COVID-19 restrictions PA is arguably more important than ever, especially during periods of self-quarantine and self-isolation [13, 14]. In addition to mental well-being PA plays an important role in immunology [15], and indeed these benefits to the immune system extend to respiratory infections such as COVID-19 [16]. Throughout the pandemic, digital platforms have emerged as a potentially promising way to engage in PA while abiding by public health regulations [17]. Online searches for topics such as ‘home-based exercise’ and ‘High-Intensity Interval Training’ (HIIT) peaked in the first two weeks after restrictive public health recommendations were instituted [18]. Several studies have also recommended the use of online exercise as a measure to increase levels of PA during COVID-19 [10, 12, 19]. However, research is still unclear if online PA interventions are as effective and acceptable to the population as in-person interventions.

Similarly, medical education curricular initiatives were required to engage students in online learning via digital platforms due to the restrictions on in-person teaching at universities. Understanding the difference between online and in-person learning is critical when shifting programmes into an online format [20]. A systematic review identified several factors important for online learning, such as interaction and collaboration, however, the impact of these factors on students remains uncertain [21] with research lacking on combined educational and experiential curricular interventions.

This study examines an exercise intervention delivered as part of a medical school’s curriculum – the ‘MED-WELL’ programme [7]. In 2020 the ‘MED-WELL’ programme was delivered in-person, however due to the COVID-19 pandemic the programme was delivered online in 2021. This presents a unique opportunity, and natural experimental context, to compare participant experiences of the same exercise intervention when offered online versus in-person. This natural comparison group will help to reveal and understand differences in experiences, and highlight how the ‘MED-WELL’ programme changes with the introduction of an online mode of delivery. The aim of this research is to optimize the ‘MED-WELL’ programme for medical students by investigating the experiences of programme participants when it is offered online versus in-person.

## Materials and methods

### Study design

This study is a qualitative study carried out using semi-structured, one-to-one interviews. The consolidated criteria for reporting qualitative studies (COREQ) was used as a guideline in all domains of the study [22]. The COREQ 32-item checklist consists of three domains: research team and reflexivity, study design, and analysis and findings. The checklist was reviewed and completed over the course of the study to help ensure consistent and thorough reporting while also improving the comprehensiveness and rigour of the study.

### Setting

This study was conducted in a single graduate-entry medical school in Ireland.

### Context

This study investigates experiences of participants in the ‘MED-WELL’ programme, an exercise and education programme delivered as part of a medical school’s curriculum in year two of a four-year graduate-entry medical programme. As part of a mandatory curricular module, all year two students are given the option of participating in the ‘MED-WELL’ programme or a mindfulness-based programme of the same duration. The ‘MED-WELL’ programme consists of eight weekly sessions, each integrating an experiential component of exercise and an educational component of prescribing exercise as medicine in clinical practice; the development of the programme is described in more detail elsewhere [7] and a brief outline is described below.

The ‘MED-WELL’ programme was originally developed to be delivered in-person and in 2020 was held in an indoor sports arena. At the beginning of each session a PA and health professional spoke for 20 minutes to the participants on an exercise or health-related topic. The related slides for each talk were made available for the students via an online learning management system prior to each session. An hour of each session was then spent engaging in different types of instructor-led, in-person PA. The remaining time was then used to complete a weekly reflective assignment as part of the curricular requirements. All eight sessions were held on Monday evenings from 4:30 to 6:00 PM.

The ‘MED-WELL’ programme was re-formatted in 2021 due to COVID-19 restrictions and was delivered online using the video meeting platform Zoom. Students could engage from a laptop computer, tablet device, or mobile phone in the environment of their choice. Students were encouraged to have their camera on however it was not mandatory. They were also asked to keep their microphones muted to limit session disruption. At the beginning of each session a PA and health professional spoke to the participants for 20 minutes on an exercise or health-related topic. An hour of each session was then spent engaging in different types of PA, led by live on-camera instructors. Participants were asked to complete a weekly reflective assignment in their own time. All eight sessions were held on Wednesday mornings from 8:00 to 9:30 AM.

### Recruitment and participants

Study participants comprised two groups of individuals: in-person participants and online participants. All graduate-entry medical students who participated in the ‘MED-WELL’ programme in-person in 2020 (*n* = 70) or online in 2021 (*n* = 89) were invited to participate in the study.

Recruitment was conducted via emails to each participant of the ‘MED-WELL’ programme informing them of the study. Programme participants were also asked to inform their peers of the study as well.

Study participants were chosen using a convenience sampling method. Sampling continued until data saturation had been reached, meaning the data that had been collected and analyzed was sufficient to address the research question while also offering a variety of participant experiences.

### Data collection

One-to-one interviews were used for data collection. The interviews were conducted by one member of the research team (AW) during February–April 2021 online using Microsoft Teams. The interviewer was a female graduate-entry medical student who had previous experience in quantitative and qualitative research regarding the ‘MED-WELL’ programme [7]. Interviews were based around 12 interview questions, developed by the research team, in the context of the research question [23]. There was a slightly different version of the questions for each cohort reflecting the differences in online versus in-person delivery (see Additional file 1).

Each study participant was emailed their respective version of the interview questions two days before the interview with the intention of giving study participants time to reflect on their answers. The interviews ranged from 23 to 39 min and were digitally transcribed by the Microsoft Teams software. The interviewer also took notes throughout each interview to help track the discussion and encourage deeper interviewee reflection. The digital transcriptions were individually reviewed by the interviewer and compared with their respective original interview audio to ensure complete accuracy. Transcripts were then anonymized but had identifiers of online/in-person, and male/female participants.

### Data analysis

#### Theoretical framework

The theory of online learning was identified as a theoretical framework during the process of data analysis, adding depth and rigour to the thematic approach in line with Varpio et al.’s discourse on the use of theory in data analysis [24]. This theory considers different forms of interaction which are integral in the educational context: interaction between participants, between participants and instructors, and between participants and the educational content [25]. The theory of online learning was further expanded in 1997 by Burnham and Walden to include participant interaction with the environment as well [26]. This theory was identified during data analysis as the ‘MED-WELL’ programme is a curricular initiative with both educational and experiential objectives.

#### Coding

A team of five researchers (AW, AOR, MC, PH, LG) analysed the data following the principles of inductive thematic analysis and based on the six steps outlined by Braun and Clarke [23]. To facilitate data analysis the transcripts were uploaded and coded using NVivo (version 12). Initially three transcripts were randomly chosen and circulated to two researchers to code independently. Two of these were from online participants and one was from an in-person participant. These three transcripts were coded independently with a subsequent meeting to review and agree on codes.

#### Thematic analysis

After the initial three transcripts were coded, another seven transcripts were circulated to the two coders. This was followed by a meeting with the two coders to ensure consistency and agree on codes. At this point one half of the transcripts were coded with an associated 250 codes. The remaining 10 transcripts were then coded with the research team available to meet if there were any uncertainties. After coding all 20 transcripts there was a total of 540 codes. After secondary coding there were 206 distinct codes, each with an individual definition that formulated the basis of a codebook. This stepwise process of coding the data involving a number of researchers ensured a rigorous approach to the analysis.

The codes were presented by two coders to all members of the research team on Microsoft Teams. To ensure the awareness of reflexivity in that one’s own beliefs can affect interpretation the research team reviewed all the data and contributed to the thematic analysis [27]. The goal of the meeting was to familiarize the research team with the data and the organization of the codes to help facilitate a shared understanding of its meaning. The codebook was circulated to the research team prior to the meeting, with a unanimous agreement that no new codes were emerging. The meeting highlighted certain codes with associated definitions and illustrative quotations from the transcripts. Through a process of discussion and iteration, codes were merged into higher level codes. Recurring terms were identified and understood in the context of the research question.

The research team collectively agreed on the applicability of the theory of online learning and initially categorized the higher-level codes into themes [28]. The themes were compared for relatedness and further grouped into overarching themes. This approach allowed a more complex analysis of higher-level codes to higher connecting and conflicting ideas in the data.

## Findings

### Participants

Ten participants of the in-person ‘MED-WELL’ programme were interviewed on their experiences, three of whom were male. Ten participants were interviewed on their experiences of the online ‘MED-WELL’ programme, three of whom were male as well. The age of study participants ranged from 25 to 34 years.

#### Overview of findings

**Fig. 1:**
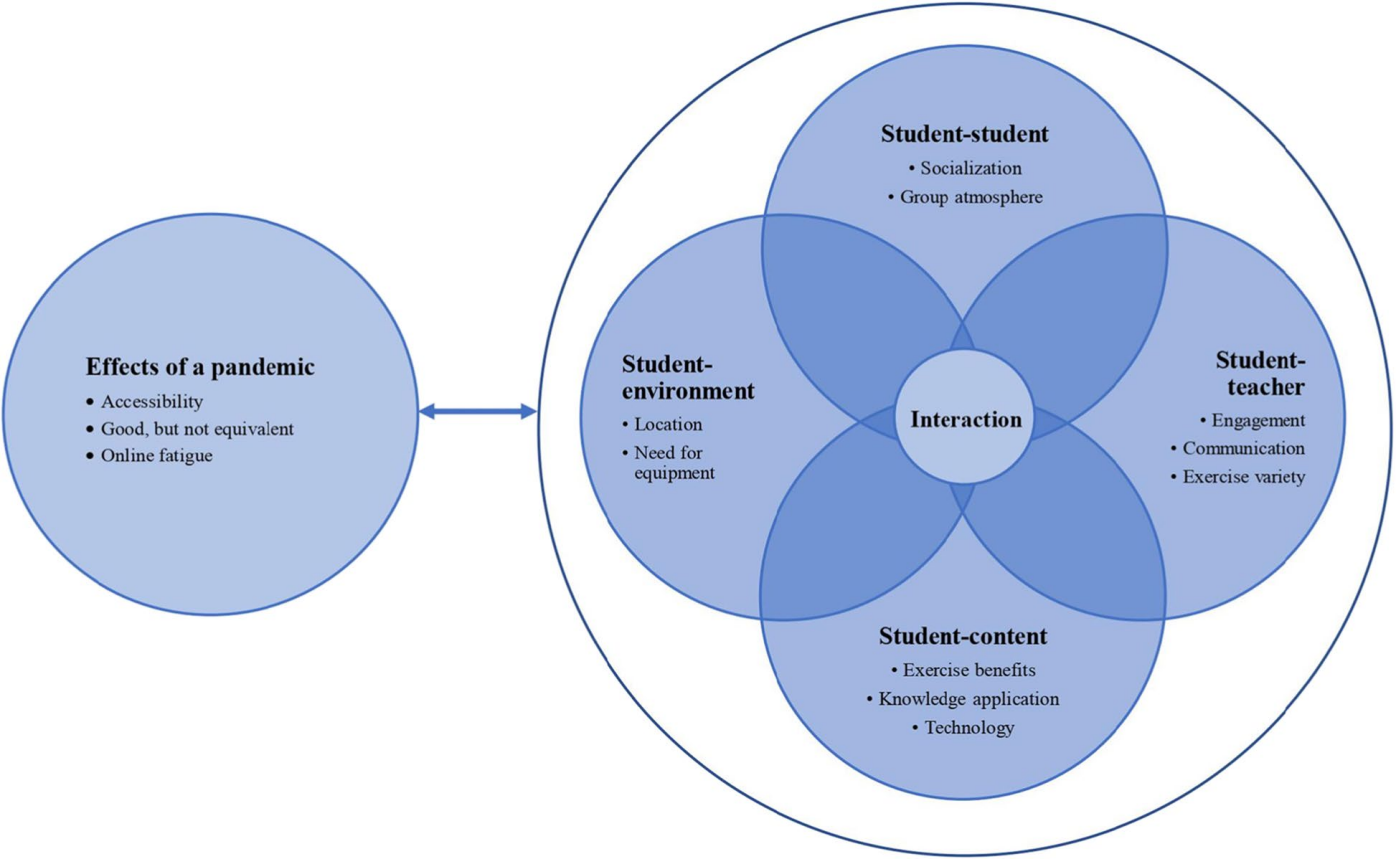
Interrelations of themes and subthemes

The data analysis produced five major themes: ‘student-student, ‘student-teacher’, ‘student-content’, ‘student-environment’, and ‘effects of a pandemic’. Each theme contained subthemes that that, at times, interconnected between major themes (Fig. 1). The findings below place the themes in the context of the above-mentioned theory of online learning which proposes different modes of interaction that influence online learning. Participants were identified as either participating in the ‘MED-WELL’ programme in-person (IP) or online (OL).

In this study the natural difference in the online versus in-person ‘MED-WELL’ programme was how it was offered and how students interacted with the various components. It was also important to consider the context of which this research was conducted – during the COVID-19 pandemic – and the effect of such context.

#### Student-student

The theme of ‘student-student’ encompasses the interactions between programme participants or more appropriately, between fellow medical students. It occurs both online and in-person via one-to-one, small group, and large group interaction. Within this theme lies two distinct subthemes of ‘socialization’ and ‘group atmosphere’, both of which play a role in motivating and engaging students.

##### Socialization

A recurring theme in almost all participant transcripts was the idea of socialization and the ability and ease of which participants could socialize with each other. When asked what in-person participants enjoyed the most about the programme, they often discussed the ability to both socialize and exercise. This social interaction was commonly missed by online participants. Two online participants mentioned how their experience solidified their thought that being able to socialize and exercise offers another dimension of engagement to the programme – a dimension that was lost in the online mode of delivery. One online participant referred to the social interactions as “micro interactions”, and spoke about the personal effects of missing out on them. Another online participant referred to the fact that the socializing aspect of exercise was motivating, and when the exercise programme was delivered online, they felt the motivation to exercise was “just gone”.

“I think I'd prefer going out and doing it socially because if I was doing it here, it would just be a home workout and I wouldn't really be able to see or interact with anybody really.” [Participant 10-IP]

“I did enjoy the program but I would have enjoyed it even more if there was a social aspect to it.” [Participant 8-OL]

Although the majority of participants highlighted the importance of combining socialization with exercise, not all participants felt that socialization was a necessary part of exercising. One online interviewee highlighted the idea of sport versus exercise, and that socialization is necessary for sport but not for exercising in the context of the ‘MED-WELL’ programme.

“I don't think it's necessary. If it was a sport, I would say maybe, but I think for me when it was just exercising, that wouldn't have been something I associate with socializing anyway.” [Participant 6-OL]

##### Group atmosphere

The subtheme of group atmosphere delineates the effects of exercising while having peers physically around you. For many participants a group atmosphere created a sense of camaraderie and helped foster the building of relationships, alongside creating a sense of accountability for participants to both attend the exercise sessions and engage in them. Some of the in-person participants discussed this in the context of what would be missing in an online environment, as opposed to what they enjoyed about their own experience.

“I love just being face to face with people. I feel like that's part of the experience. You know sometimes you lose that with online platforms and it kind of takes away a little bit of the excitement.” [Participant 5-IP]

“If I was involved in something in-person there's a good chance that a couple of my colleagues and I might get a bit of a relationship going there and do something together in-person in the future.” [Participant 2-OL]

Social pressure was a recurring dimension of this subtheme, often relating to how the group atmosphere influenced participant engagement. Differing opinions emerged regarding social pressure, as it was a motivating factor for some participants but was a deterrent for others. For some participants the social pressure was motivating in that they did not want to be embarrassed in front of their peers. This tied into the idea of competitiveness, which a few other participants mentioned was a driving factor in how the social pressure motivated them. Conversely, some participants preferred the lack of a group atmosphere online, in that there was less social pressure. The idea was that online exercise could limit perceived feelings of self-consciousness and embarrassment that might come with in-person exercise.

“I suppose it helps like that because you're surrounded by your peers. You know, there is that pressure that you do need to give it everything, to not be embarrassed.” [Participant 8-IP]

“I didn’t feel like I was the uncoordinated, sedentary one in the corner.” [Participant 6-OL]

Many participants related their preference for a group atmosphere to the idea of being an extrovert, and similarly the potential for those drawn to online exercise having that of an introverted personality. The majority of participants who made these types of comments referred to themselves as extroverts, and inferred that the opposite would be true for introverts.

#### Student-teacher

This theme encompasses the interactions between participants and the teachers in the ‘MED-WELL’ programme, which includes both instructors for exercise and lecturers for educational sessions, and how those interactions influenced participant experiences. Both in-person and online participants contributed feedback on the exercise instructors and educational lecturers themselves with an undeniably positive regard. Within this theme are three subthemes: ‘engagement’, ‘communication’, and ‘exercise variety’.

##### Engagement

Many of the personal attributes of the instructors and lecturers contributed to how well participants engaged in the programme both in-person and online. Attributes such as encouraging and entertaining were mentioned for exercise instructors, and well-educated and enthusiastic for educational lecturers. In-person lecturers were often credited for making the lecture “interactive” by asking questions and opinions throughout the lecture.

Different learning styles were apparent when engagement was discussed in the context of the educational lectures. Online lecturers had the option of including visual slides during their presentation. Some online participants preferred the use of slides, whereas other did not. One online interviewee mentioned it was more engaging when the lecturer spoke extemporaneously.

“The lectures, you know a lot of the time they ask questions and they're expecting responses, it was very interactive. And I could stay engaged because it was more interactive.” [Participant 7-IP]

“I think that’s nicer sometimes to engage with people that are talking about something that's kind of fluent and they parlet, as opposed to something that they put together, there’s loads of slides, here’s the information.” [Participant 1-OL]

##### Communication

How participants interacted with instructors and lectures often related to the quality and context of communication with participants, in the form of both giving knowledge and feedback. Exercise instructors in particular were seen as providing good explanations of the exercise; this clear instruction also helped participants engage in the exercise session.

Both online and in-person participants mentioned how it was helpful that instructors would also participate in the exercise to demonstrate the movements. Online participants seemed to find this easier with a screen in front of them, whereas a few in-person participants found this was more difficult in-person because they could not always see the instructor.

“A lot of the instructors would continue to do the movements throughout the exercise, so if you did get stuck or if you fell behind or kind of forgot what you were doing, you could always look at them and figure out how to do it.” [Participant 8-IP]

A few of the online participants noted the lack of personal feedback that existed in an online setting because instructors could not physically see each participant, whereas in-person instructors could walk around and observe participants. One interviewee noted that even if participant cameras were turned on, the opportunity for feedback is difficult.

Risk of injury was noted on more than one occasion as a consequence to this limited feedback. If instructors were not able to observe a participant’s form there was a risk that participants could perform the exercises incorrectly and injure themselves, either during the session or over time. One participant noted the “domino-effect” that this could have: if the ‘MED-WELL’ programme is meant to teach medical students about types of exercise to prescribe them in clinical practice, the impact of learning them incorrectly initially could carry into the instruction of future patients. The use of cameras online interrelates to the subtheme of ‘technology’ within the theme of ‘student-content’.

“They couldn’t give immediately feedback in terms of like if you were doing it wrong, or doing the exercise with poor form, you wouldn't really address that.” [Participant 5-OL]

“I think sometimes exercise programs delivered online aren’t the best because they could precede injuries in the sense that like the person can't see you doing it.” [Participant 7-OL]

##### Exercise variety

When asked what participants enjoyed about the ‘MED-WELL’ programme, variety of exercise was a recurring answer. Almost all of the in-person participants were satisfied with the variety that was offered in their respective classes. One female participant felt there was repetition in a few of the sessions. Often participants would highlight a particular type of exercise they found enjoyable such as a yoga or boxing class. A variety of instructors also complemented the variety of classes.

“I liked that there was a mix between the weeks of high intensity and then yoga.” [Participant 8-OL]

“There were no two evenings I came out of the class saying that it was similar to another evening we had. It was good.” [Participant 6-IP]

Online participants seemed less satisfied with the variety that was offered, and acknowledged that the variety of online classes is “limited”, not a fault of the instructors but as a result of the space and lack of equipment. This interrelates to the subtheme of ‘need for equipment’ under ‘student-environment’. A few online participants spoke about their satisfaction with the variety, but how it is easier to have a greater variety in-person.

“I kind of understand that for them [instructors] it's not that easy to come up with that many training sessions with no equipment at all.” [Participant 4-OL]

#### Student-content

As the ‘MED-WELL’ programme incorporates both an educational and experiential aspect, this theme embodies how participants interacted with both the educational lecture and the exercise components of the programme. Three subthemes emerged from this theme: ‘exercise benefits’, ‘knowledge application’, and ‘technology’.

##### Exercise benefits

What participants felt they “got out of the exercise” is the heart of this subtheme. The majority of all participants enjoyed the exercise content and how it made them feel. A few participants noted the physical effects that exercise had on them, such as better sleeping, while others highlighted the mental health effects. These positive benefits did not seem to change with the mode of delivery of the exercise.

“If you had a stressful day or a bad day, it was stress release with the exercise, and then it was like “oh I feel great now.” [Participant 4-IP]

“You felt like you've done something and it was kind of a nice post-exercise feeling, like after going to bed you felt well-rested that night.” [Participant 9-IP]

Participants from both online and in-person sessions also described their interest and action in continuing to exercise after the programme was completed. Two in-person participants mentioned continuing a specific type of exercise – weight-training and HIIT. One online participant stated how the weekly session encouraged them to be active more times throughout the week. Another online participant found the sessions helpful in discovering ways to exercise online when COVID-19 limited other exercise activities, which interrelates to the theme of the ‘effects of a pandemic’.

“I’ve done a few [online classes] since, and I actually think I never gave it enough of a chance…once I had my little space and my mat I enjoyed it a lot more than I expected.” [Participant 6-OL]

##### Knowledge application

One of the objectives of the programme overall was to educate participants on the concept of exercise as medicine in clinical practice. The consensus amongst all participants was that the educational sessions offered content that they could potentially use in their future career as a doctor. This aligned with the fact that the majority of the lecture content surrounded the idea of prescribing exercise as medicine to future patients. For some participants this content was new knowledge, and for others the content solidified their existing knowledge. Many interviewees relayed a new-found confidence in their own ability to integrate exercise into clinical practice.

“It just solidified my beliefs and made me really that much more pumped and passionate about these types of programs.” [Participant 5-IP]

“I personally would have known a good amount about nutrition and exercise already, so it's like I don't think I learned anything ground-breaking.” [Participant 10-IP]

The lectures seemed to provide a sufficient science-based foundation on different exercise topics, which participants connected to their existing practical knowledge of exercise. Participants found this connection created a sense of authenticity to their knowledge of exercise as medicine; this sense of authenticity was one of the factors that seemed to influence participants’ confidence in their own ability to integrate exercise into clinical practice.

“There's a lot of things that came up that I had thought of, but I didn't have any basis to back it up, or no one in the medical world had told me that.” [Participant 3-OL]

“I would have no trouble at all promoting this as, let's say, a GP to my patients. You know if they were struggling to get involved in an exercise program, or struggling to lose weight, or trying to get their blood pressure down.” [Participant 5-IP]

##### Technology

Participants often referred to the use of technology in the context of it being a barrier to engaging with the programme content. Interestingly the majority of participants who acknowledged technical difficulties were in-person participants, commenting on difficulties with the speaker and microphone. The majority of online participants did not have technological difficulties, with only two having issues with internet connection to the online video meeting.

There were consistently different opinions on the idea of turning personal cameras on when exercising online. Some participants supported the idea of having cameras on as it would help encourage engagement. Another group of participants were reluctant to turn on their cameras, and felt more comfortable and less self-conscious with their camera off. All participants who expressed a desire to keep the camera off were female, whereas those with an interest to encourage camera use were both male and female.

It was not mandatory for participants to have their camera on during online sessions, and a few interviewees from both groups suggested that this could lead to higher level of disengagement; some participants put it as “opting-out” of exercising. This concept interrelates to the idea of motivation, as without a group or an instructor it was easier to disengage from the exercise session.

“It does make you feel like you're all in the room together and you all have to participate.” [Participant 7-IP]

“You know when you turn video off, turn the microphone off and you're just zoned out and you're not taking part.” [Participant 8-IP]

“You probably could engage less, like you could give up more easily when you're just doing it off the screen and you're not participating in a group.” [Participant 7-OL]

#### Student-environment

This overarching theme explains the interaction of the participants with their environment and surroundings. It also details how that interaction affected participants’ ability to engage and their overall enjoyment of the programme. This theme includes two subthemes relating to infrastructure: ‘location’ and ‘need for equipment’.

##### Location

There was a diversity amongst opinions on how the programme location affected participant experience. Some in-person participants appreciated the larger exercise space whereas another participant felt it was too small. The large gymnasium location sometimes hindered interaction by making it difficult to hear exercise instructions and lecture material. This meant participants were not able to fully engage in the session and were instead focusing on trying to catch-up on the exercises.

“The setting was really good, I think it was big enough to accommodate us all.” [Participant 8-IP]

“I remember it was a bit confined, the space. So I think the day we were doing yoga or Pilates, we were all on top of each other.” [Participant 7-IP]

The online environment was often an at-home space, usually the participant’s bedroom or living room. Some people quite enjoyed exercising at home; one female participant credited having a good technological setup as what influenced her positive experience. This paralleled feedback from another participant who mentioned their video setup made it a bit more difficult to engage.

“The only difficulty I can see is just when we're doing it, kind of in our rooms, just the placement of the video versus us on the floor, it’s a bit to coordinate.” [Participant 9-OL]

One major influence that location had on participants was on time spent travelling to the programme session. Some in-person participants found no issues with travelling as the sessions were on-campus and participants were able to walk. From a scheduling perspective other in-person participants expressed feeling rushed having to travel across campus. However, these participants often noted they had other curricular commitments before and/or after the ‘MED-WELL’ programme which contributed to their negative feelings towards the in-person location.

“In the afternoon, you know, you kind of already had a full day and I found myself rushing from my studies.” [Participant 5-IP]

Online participants often mentioned that the lack of travel time was a benefit of having an at-home location. From a scheduling perspective less time had to be set aside to exercise, which meant that online exercise was more accessible for participants. In fact, the lack of travel time is what some participants felt was the greatest benefit over in-person sessions. As well, one online participant mentioned that with online exercise she was able to take part in exercise more frequently because she did not own a car.

“I think there is a role for them, especially for people who can't make it out, don't have that option.” [Participant 10-IP]

“It probably made it easier for some people, I suppose who weren't living in Limerick but they were able to go online. But just the quality obviously went down…There’s definitely an opportunity there, but for teaching classes or teaching medical students, it definitely it should be done in person.” [Participant 5-OL]

##### Need for equipment

This subtheme arose from data surrounding the use of exercise equipment and participants’ ability to engage with different exercises, mostly pertaining to the potential limitations of the online environment. Interestingly it was the in-person participants who discussed the need for equipment and inferred that it would be more difficult to engage in exercise without the use of different equipment, such as kettlebells, dumbbells, or yoga mats. A female participant relayed that she enjoyed in-person more because it did not require her to have any equipment of her own. An added benefit of equipment was also the opportunity to alter the difficulty of different exercises.

“I think it works better because we have the equipment for it. I think it would be difficult to do if you didn't have the equipment.” [Participant 4-IP]

“I didn't have a yoga mat and I just wasn't really prepared for online. Having access to it [the ‘MED-WELL’ programme] was great.” [Participant 2-IP]

Although the majority of attitudes on exercise equipment were heard through in-person participants, one male participant appreciated that online sessions helped him learn new exercises without the use of equipment. In contrast, another male participant noted how the purpose of the online exercise itself was changed because of the lack of the equipment.

“I've never really done exercise without weights before…I completely forgot how to workout without weights. So this kind of showed me that there is a way to exercise without weights.” [Participant 10-OL]

“In the very first session we did a strength session, and it just it ended up an aerobic session because nobody had any equipment. I think everyone was at home and there was nothing really you could do about it.” [Participant 5-OL]

#### Effects of a pandemic

This theme relates to the context of why the ‘MED-WELL’ programme was offered online. Many participants referenced the current climate of the COVID-19 pandemic in how it affected their ability to exercise, and their appreciation for different modes of exercise delivery. It consists of three subthemes: ‘accessibility’, ‘good, but not equivalent’, and ‘online fatigue’.

##### Accessibility

Both online and in-person participants acknowledged how accessibility of exercise changed during COVID-19. The closing of gyms and leisure centres was repeatedly mentioned, and some individuals felt that they became less active as a result of these closures. This in turn led to an appreciation for online exercise in a climate where in-person exercise was not accessible. Indeed, when the ‘MED-WELL’ programme was initially offered online, it seemed to be more enticing as exercise options were limited at the time.

“I know being in lockdown, like I've been doing a lot of stuff online because there's nowhere else you can do it. You can't go to a gym.” [Participant 2-IP]

“Near the beginning of the year I was very not active, like the gyms are closed.” [Participant 10-OL]

##### Good, but not equivalent

As one online participant put it, “online sessions are better than no sessions”. A few online participants felt the online sessions were acceptable simply because it offered a form of exercise when exercise options were limited. Many online participants commented on their preference to exercise online rather than not exercise at all, and delineated a middle-ground where online exercise was superior to no exercise, but still inferior to in-person exercise. When asked about this general inequality of in-person and online modes of delivery, many participants had the opinion that they could never be the same.

“I think it's always better to be in person…I think if I have to do it online, in this kind of case, the reason would be because there's no other option.” [Participant 10-IP]

“It's hard to find negatives with it because there's no alternative. This [exercise] doesn't happen without the online.” [Participant 2-OL]

“I think there is a place for it [online exercise program]. But again it will never replace, I don't think it could ever replace, in-person.” [Participant 4-OL]

Interestingly, no participant shared a steadfast view that online was superior to in-person exercise. However, a few participants discussed how the COVID-19 pandemic created a circumstance in which exercise should be offered online, despite its perceived inferiority.

“I don't think it should be delivered online, but I understand of course why it is... like if COVID wasn't around I would expect it to be in the gym.” [Participant 10-OL]

##### Online fatigue

A concept of online fatigue emerged from several of the discussions with online participants and one in-person participant. Overall, online participants were tired of constantly being online as the pandemic forced many activities to be delivered that way. Exercise, medical school lectures, and socializing were all mentioned as having online components; positive feedback towards online sessions was sometimes overshadowed by this fact.

“I definitely think there is a place for them, but it is tiring when you're doing that only, all day… I do feel a lot more tired at the end of the day than I would if everything would be in person.” [Participant 4-OL]

“I think it's hard, because if you had asked me this in January or February I’d be still really for it [online]. But I think we’ve all just reached a wall now.” [Participant 3-OL]

When discussing online fatigue participants naturally included the negative effects of the pandemic on their mental health overall. One online participant felt she was more mentally tired at the end of a day of online activities, compared to when they were in-person. A few online interviewees relayed that it was a “tough year”, with one participant in particular indicating their own social relationships had suffered due to the lack of in-person contact.

“I would say I don't necessarily love this whole ‘zoom year’ we've had and ‘virtual year’. It's been tough on me. I know I'm not alone.” [Participant 5-IP]

## Discussion

### Summary of main findings

The data from this study outlined a range of diverse opinions amongst participants regarding the research question. Interestingly opposing opinions were not always those of online participants versus those of in-person participants. Differing opinions seemed to be the result of personality differences, previous exercise experiences, and personal preference; intra-group differences were often more prominent than inter-group.

The in-person ‘MED-WELL’ programme allowed socializing and a group atmosphere that seemed to be important for motivation and engagement. The in-person programme also offered a greater variety of exercises, in-part related to the availability of exercise equipment. Conversely, the online ‘MED-WELL’ programme was deemed more accessible as there was no travel time, and despite an overarching view that online exercise is inferior to in-person exercise there was a general consensus that online exercise is better than no exercise at all.

Interestingly, certain factors that were motivating for some participants were deterrents for other participants to engage. Some of these factors were the social pressure in a group atmosphere, and the use of cameras during online exercise.

The mental and physical benefits of exercise did not seem to vary between the mode of exercise delivery. Similarly, participants of both online and in-person sessions were able to engage and apply the educational lecture content. In both modes of exercise delivery, the quality of the instructor communication was an important factor in engaging in the sessions.

Common to all five overarching themes was the key concept of motivation. Participant interaction with other students, teachers, the programme content and the environment impacted how they were able to engage with the ‘MED-WELL’ programme and their overall enjoyment of the programme itself. The onset of the COVID-19 pandemic also had an evident impact on the experiences of the online participants, highlighting a realization of the potential advantages of online programmes but also the drawback of overuse.

### Comparison to existing literature

Even prior to the onset of the COVID-19 pandemic, lack of motivation was viewed as a barrier to engage in an online setting [29]. When delivered online, other medical school education programmes have shown similar feedback such as difficulty engaging in content, and lack of socialization. The lack of exercise equipment in an online mode of delivery was also a barrier to engagement, which has been seen in other research as well [30]. On the other hand, feedback is consistent regarding better accessibility and lack of travel time with online programmes [31–33]. Other studies have examined the effectiveness of online medical education generally and have supported the idea of a blended teaching method, combining online and in-person interactions [34].

Interaction is generally seen as a critical component to the educational process [25] and indeed much of the study findings revolve around how participants interacted with various entities of the ‘MED-WELL’ programme. The importance of participant motivation was apparent throughout the thematic analysis. Indeed motivation has always played a strong role in participants engaging in online learning [29]. Motivation is also a widely studied concept in exercise, both in defining behavioural theories and modelling behavioural change [35–37]. By understanding what motivates behaviours of individuals, programmes and interventions can be optimized and better targeted towards the needs of participants.

There are several relevant frameworks that attempt to explain motivation in exercise, with many emphasizing the social dimension and context of participation [38]. The social aspect of exercise has been found to be a common motive for exercising and important for overall enjoyment of exercise [39, 40]. This is magnified in the context of the COVID-19 pandemic as well; many participants mentioned missing the social aspect in an online mode of delivery. This complements current research that highlights the positive impact that social support has on exercise engagement, specifically during the COVID-19 pandemic [41].

A few online participants mentioned the idea of ‘online fatigue’; a sense of tiring of online activities due to their pervasive nature during the COVID-19 pandemic. This is an emerging concept from the pandemic, with the trending term ‘zoom fatigue’ to reflect the commonly used video meeting software [42, 43]. Similar to our study findings on participants ‘opting out’ of online exercise and finding it difficult to focus, other research has found communication and participant attention issues with overuse of online activities [44].

The attitude of many online and in-person participants was that they enjoyed the exercise content and how it made them feel, commenting on positive mental and physical effects on their well-being. This finding reinforces the well-studied role of exercise in both mental and physical well-being [45]. It also aligns with previous quantitative research on the ‘MED-WELL’ programme which demonstrated the positive effects on participants’ overall well-being [7].

Almost all study participants discussed ways that they would apply content from the educational lectures in a clinical setting. Many conveyed a sense of having gained knowledge during the programme, with the majority confident that they would carry that knowledge forward into their clinical career. These findings are contrasted against current research, which still shows a deficit in medical professional knowledge in exercise as medicine [46, 47].

Another key topic was that of exercise location, with the online experience revealing a sense of greater accessibility to exercise for some participants in terms of less travel time and a lesser feeling of self-consciousness. This is especially important in the setting of the COVID-19 pandemic where there was an evident increase in the use of online exercise resources [12, 18]. Interestingly, the feelings of self-consciousness were expressed by female study participants only, aligning with the previously studied concept of self-conscious emotions impacting exercise specifically in females [48].

Many of the above-mentioned findings are seen in other literature depicting motivations and barriers to exercise. An appreciation of these findings demonstrates how to optimize the ‘MED-WELL’ programme for future delivery to medical students, be it online, in-person, or through a blended teaching method.

### Strengths and limitations

This study involved a multi-disciplinary research team undertaking a rigorous and step-wise analysis of the qualitative data using two comparison groups. Having two comparative groups enabled an analysis of experiences in different contexts and helped to identify different phenomena and how they differ between groups [49]. The data sample was also large (*n* = 20) and achieved data saturation.

This study was conducted in one medical school so findings may not be generalizable to other settings. By using a convenience sampling method the interview groups were not adjusted to account for gender differences, although the female to male ratio of each sampling group closely reflected that of the overall medical class. Participants volunteered for the study, and the interviewer was also a colleague to some of the study participants which could have generated a response bias. Interviews for the online group were conducted shortly after participation in the programme whereas interviews for the in-person group were conducted 10 months after programme participation, which could contribute to a recall bias.

### Implications for future research and practice

With the trend of shifting in-person activities online due to the COVID-19 pandemic comes the inevitable decision of how to move forward when in-person activities can safely be offered again. This study yielded some interesting and noteworthy results, and helped to expose both weaknesses and strengths of the ‘MED-WELL’ programme when offered online and in-person. One-to-one interviews proved to be invaluable for conveying reasoning behind participant experiences and opinions.

One aspect of the ‘MED-WELL’ programme was experiential; having participants engage in PA to promote their own health and well-being. Both in-person and online participants conveyed positive mental health effects from the programme, with some indicating a continued interest in exercising after the programme had completed. The second aspect of the ‘MED-WELL’ programme was the educational component, and teaching medical students about prescribing exercise as medicine in clinical practice. Almost every study participant conveyed a sense of having gained knowledge during the programme, with the majority confident that they would carry that knowledge forward into their clinical career.

Overall, the optimization of the ‘MED-WELL’ programme comes down to accessibility and personal motivation to engage in the programme itself. The ability to adapt the programme to suit individual preferences on elements such as the mode of delivery, type of exercise, and timing could enhance the programme overall. This reflects the impact that a sense of personalization could have on the programme, and complements the idea that autonomy in exercise can positively affect exercise engagement [50]. Future research should take into account underlying exercise experience of participants.

## Conclusions

The findings from this study have highlighted the importance of participant motivation and engagement for the success of education and exercise-based programmes. The ability to engage in a satisfying and meaningful way depends on personal and social factors not necessarily limited to the context of the COVID-19 pandemic.

The ‘MED-WELL’ programme remains a component of the medical school curriculum with findings from this study influencing the delivery of the programme in upcoming years. The findings from this study can also be used to inform similar experiential and educational PA interventions, and may help plan for potential future restrictions on in-person activities.

## Supplementary Information

**Additional file 1.** Interviews questions for online and in-person participants.

## Data Availability

All relevant data is published in the paper as illustrated quotes. For inquiries regarding data supporting the findings presented in the manuscript, please contact the corresponding author.

## Abbreviations

PA: Physical Activity
WHO: World Health Organization
HIIT: High-Intensity Interval Training
