# Supplementary material for: Lessons learned from a pandemic: implications for a combined exercise and educational programme for medical students
Source: BMC Med Educ. 2022 Apr 8;22:255. doi: 10.1186/s12909-022-03290-1 (PMC8990684; doi:10.1186/s12909-022-03290-1)
Supplement: Supplementary file 1 — Additional file 1. Interviews questions for online and in-person participants. [file 12909_2022_3290_MOESM1_ESM.pdf]

UNIVERSITY OF LIMERICK
OLLSCOIL LUIMNIGH
School of Medicine

Interview Questions

Title of Study: Lessons learned from a pandemic: implications for a combined exercise and educational programme for medical students

Note: Online/face-to-face** options are different based on interviewee group*

1. Why did you choose the 'MED-WELL' programme over the mindfulness programme?
2. What components of the 'MED-WELL' programme did you find most enjoyable and why?
3. What components of the 'MED-WELL' programme did you find least enjoyable and why?
4. Were there any limitations in taking part in the 'MED-WELL' programme *online*/face-to-face***?
5. Were you able to thoroughly engage in the interactive lecture and physical activity of each session? Can you tell me more about this?
6. What did you learn from the sessions that you could implement into your own medical practice?
7. How would you describe the content (variety of classes) and the standard of training provided?
8. Could you tell me how easy or difficult you found it to follow the instructions and why?
9. What were your expectations of *an online*/a face-to-face*** exercise programme and did the 'MED-WELL' programme match those expectations?
10. What are your views on online group activities in general (for example, work meetings, lectures, or socializing)?
11. Do you think an exercise programme *is suitable to be delivered online*/can be delivered online*** and why?
12. What recommendations do you have for improving the 'MED-WELL' programme?
